# Bullying within the Forestry Organizations of Turkey

**DOI:** 10.1155/2013/851839

**Published:** 2013-06-15

**Authors:** Devlet Toksoy, Mahmut Muhammet Bayramoğlu

**Affiliations:** Karadeniz Technical University, Department of Forest Faculty, 61080 Trabzon, Turkey

## Abstract

Today, many studies are conducted in order to determine bullying behaviors and to resolve conflicts with the purpose of increasing and maintaining organizational success in developed countries. According to these studies, bullying cases are more common in public institutions when compared to other sectors. In public institutions, bullying generally occurs when successful workers are discouraged and/or harassed by their managers, thus leaving them feeling distressed and dissatisfied with their jobs. The present study examines whether forest engineers working in the seven geographical regions of Turkey are exposed to bullying behaviors, the level of any bullying, and whether there are any regional differences (*N* = 835). Through statistical analysis, a significant relationship was determined between bullying and demographic characteristics. The results of the present study were evaluated along with the results of other studies, and some suggestions were made in order to prevent bullying behaviors in forestry organizations.

## 1. Introduction

Within the last decade, researchers have begun to emphasize the significance of aggressive behavior in the workplace. The scope of aggressiveness is a broad one, ranging from violence at one end to incivility at the other. Aggressiveness in the workplace is a very important subject; however, although violence attracts immediate attention because it is more readily visible and evident, subtler forms of unwelcome behavior like bullying/mobbing or general incivility might be underestimated despite the harm they cause to both organizations and individuals [1]. Therefore, today bullying receives growing attention in organization research. Bullying in the workplace, in one form or another, has always existed. In particular since the 1990s, the frequency, intensity, and psychopathological consequences of this phenomenon in the workplace have reached alarming proportions in a variety of countries, and researchers have reported alarming findings about the negative consequences associated with bullying, both for the individuals and the organizations concerned [2]. Thus, gaining a thorough understanding of why and how bullying develops in workplaces is of utmost importance in terms of the prevention of and/or managing the problem [3]. Yet, bullying is often a misunderstood, misdiagnosed, and mismanaged behavior in work environments [4].

Researchers have shown that bullying in the workplace reduces the psychological, physical, and mental health of its victims and negatively influences the well-being and efficiency of other employees [5–9]. The symptoms reported by the victims include low self-esteem, anxiety, sleep disturbance, recurrent nightmares, various somatic problems, concentration difficulties, irritability, feelings of depression, and self-hatred [10–15]. The bullying process has negative effects on both the individual and the organization [16]. Since employees being the target of bullying frequently leads to higher absenteeism, increased employee turnover, decreased commitment, intentions to leave the organization, and reduced productivity, bullying is considered to carry a substantial cost to organizations [17] and there is a strong societal dimension to the problem as society is left to pick up many of the long-term costs, including medical treatment, work incapacity benefits, and costs associated with premature retirement [18–20]. In Sweden, each bullied worker can cost an employer between $30.000 and $100.000 USD per year [21], and in the UK, the total annual cost of accidents and ill health has been estimated to be from *£*9.9 billion to *£*14.1 billion, of which, work-related illness accounts for *£*6.2 to *£*7.2 billion. The total cost to society was equivalent to between 1.4% and 2.0% of Britain's gross domestic product (GDP) in 1995/96 [22]. In the study of Carnero et al. [23] stated Pastrana [24] it was calculated that 52 million euro was lost in work compensation as a consequence of mob behaviors in Spain in the year 2002, and [25] suggested that the direct annual cost of psychological intimidation in Germany was $112.000 and the indirect cost was $56.000 to the companies with a thousand workers.

Studies concerning workplace bullying indicate that the frequency of this phenomenon varies in different sectors and countries. In their study, Escartín et al. [26] evaluated the opinions of the workers from two different areas, Central America and southern Europe, concerning bullying, and determined that the workers from Central America were more exposed to the physical component of bullying when compared to the workers from southern Europe. Hubert et al. [27] found that 1% of workers in the financial sector suffered from mobbing in Holland, Carnero et al. [23] reported that around 5% of workers declared they had been victims of mob behavior at their workplace in Spain in 2003, and Einarsen et al. [28] conducted a study with 5288 UK employees and determined that some 5% of the respondents were exposed to severe bullying and another 3% experienced physical abuse or intimidation. These differences may result from cultural differences, development levels, masculine/feminine values and the methods used in the studies [29–31]. Bullying is a less recognized issue in developing countries, including Turkey [32]. There have been studies in the fields of health [33–35], education [36–39], ethical environments [40], and leadership [41–43] in Turkey; however, there are only a few studies concerning the general public. The present study aims to determine whether forest engineers within the General Directorate of Forestry, which is the only authority for the forests in Turkey, with a history of approximately 200 years, are exposed to bullying behaviors, the level of the bullying, and the relationship between the demographic characteristics of the participants, the geographical locations of the branches of the Directorate of Forestry they work for and bullying. A map of bullying within the branches of the forestry organization of Turkey was made possible through the use of the obtained data. 

## 2. Definitions of Bullying

 There are many different definitions of bullying in the literature [44]. Terms such as mobbing [45, 46], harassment at work [47], petty tyranny [48], victimization [49, 50], abusive supervision [51], emotional abuse [52], workplace incivilities [53], and mistreatment [54] have been used to describe and define workplace bullying. Sometimes these words are used synonymously, but sometimes they are not. As an example, the alternating use of bullying and mobbing derives from the cultural and scientific backgrounds of the researchers who tackle the problem [55]. In general, bullying is the most preferred term in English-speaking countries, including the UK, Ireland, and Australia. Other terms have been used in southern European countries, the USA, and most notably in Scandinavia and German-speaking countries, where the English-derived term “mobbing” has been widely adopted [56].

This phenomenon was first used by Konrad Lorenz to describe the behavior set of small animals exposed to dangerous situations to protect themselves against a single larger enemy [57]. Then Peter-Paul Heinemann used it in their research examining the behaviors of children at school [58], while the first researcher to use the term in relation to working life and in organizations was Leymann, who was a pioneer in studies concerning workplace bullying. He defined the term as follows.

Psychological terror or mobbing (bullying) in working life involves hostile and unethical communication, which is directed in a systematic way, by one or a few individuals mainly towards one individual who, due to the mobbing, is pushed into a helpless or defenseless position, being held there by means of the continuing mobbing activities. These actions occur on a very frequent basis (statistical definition: at least once a week) and over a long period of time (statistical definition: at least 6 months of duration) [57].

Researchers tried to define bullying using typologies of aggression such as verbal-physical-nonverbal; direct-indirect; and active-passive [59–61]. Bjorkqvist et al. [62] reported that boys use physically aggressive strategies, while girls prefer verbal ones, while Baron and Neuman [63] suggested that verbal aggression was rated as significantly more frequent in occurrence than physical aggression. Furthermore, passive forms of aggression were rated as more frequent than active forms of aggression, and direct forms of aggression were rated as significantly more frequent than indirect forms of aggression. The verbal and nonverbal communicative behaviors are often referred to as the forms in which workplace bullying is manifested [64].

Researchers have examined whether the causes and consequences of all bullying behaviors are the same under the same circumstances (whether they are homogeneous). As a result, Leymann [21] defined 45 bullying behaviors and categorized the behaviors under 5 groups, and Ashforth [65] categorized these behaviors under 6 groups. Zapf et al. [45]. determined 7 groups for these behaviors. Keashly [52] stated that these behaviors are mostly of a verbal nature and rarely include physical violence. Another research issue in respect of bullying is the cause of such behavior and the frequency of its performance or the frequency of victim exposure to such behavior. Leymann [57] stated that inadequacies in leadership practices and poorly organized working conditions, as well as low levels of morale in certain departments, were the organizational causes of mobbing (bullying), while Schuster [66] pointed to the social system and the processes of social exclusion as a potential cause of such behavior. Bjorkqvist et al. [67] stated that the status and job position of individuals, colleague envy, and lack of self-confidence were causes of bullying, while Zapf [68] stated that the organization itself, the perpetrators of mobbing, the social structures of the workplace, and the mobbing victim him/herself were the causes of such behaviors.

There are different opinions about the effect of personality and the psychological condition of the victim of bullying. Einarsen et al. [69] suggested that envy is the main reason for bullying and that a lack of self-confidence, inefficient communication, and incompatibility are also effective in this regard. Einarsen [31] also stated that the victims' anxious, aggressive, or vulnerable natures or the fact that are open-minded, sophisticated, conscientious, and very successful, is effective on exposure to bullying. However, Leymann and Gustaffson [70] argued that victims have no distinctive features and everyone can be exposed to bullying. 

The core elements of workplace bullying, which are generally agreed upon by most researchers, are frequency and duration [71]. Most researchers in workplace bullying agree that one or a couple of instances of the above verbal and nonverbal behaviors should not be considered bullying [72]. Leymann [57] suggested that the approximate duration of bullying was at least 6 months and at most 15 months; and these behaviors are exhibited consistently and systematically over a long period, with the intention of causing damage. Einarsen and Skogstad [73] reported that the bullying duration was 18 months, Salin [74] suggested that it was 2.7 years, Rayner [75] stated that it was less than 1 year, and Zapf and Gross [76] suggested that in order to call something bullying, the behavior must occur repeatedly (once a week) and over a long period of time (at least 6 months). According to Bjorkqvist et al., it is important to assert that harassment (bullying) is not initiated by the victim, but by the tormentor, in the same way that torture is started by the torturer [67]. Without a tormentor, there would be no harassment. Therefore, the primary duty of the organizations, especially the departments of human resources, is to take the necessary precautions regarding such behaviors and to combat them. 

## 3. Demographic and Geographic Data

There are many studies examining the relationship between the demographic characteristics of the victims of bullying and the levels of exposure to bullying. Hatch-Maillette and Scalora conducted a comprehensive study concerning the relationship between gender and bullying in the workplace [77]. Quine examined exposure to bullying in accordance with the age groups of research participants in the National Health Service in England and determined that the 31–40 age group was most exposed to bullying [78]. Soylu determined that people working in the public sector were more exposed to bullying when compared to people working in the private sector, and managers were less exposed to bullying than the workers [41]. 

The forestry engineer participants in the present research were asked questions regarding their demographic characteristics, such as age, gender, marital status, and education level about the duration of the professional lives, their current positions, the number of times they had changed workplace and/or the number of units/divisions they had worked in. Hypotheses were constructed, and the relationship between these characteristics and bullying was examined, and the results were compared with other studies in the literature.

Hypotheses regarding the demographic characteristics are as follows.

**Hypothesis 1**

There is a relationship between the genders of the forest engineers and their level of exposure to bullying.

**Hypothesis 2**

There is a relationship between the ages of the forest engineers and their level of exposure to bullying.

**Hypothesis 3**

There is a relationship between the marital status of the forest engineers and their level of exposure to bullying.

**Hypothesis 4**

There is a relationship between the education levels of the forest engineers and their level of exposure to bullying.

Hypotheses regarding professional life are as follows.

**Hypothesis 5**

There is a relationship between the duration of the professional lives of the forest engineers and their level of exposure to bullying.

**Hypothesis 6**

There is a relationship between the in-house positions of the forest engineers and their level of exposure to bullying.

**Hypothesis 7**

There is a relationship between the number of changes of workplace and the level of exposure to bullying of the forest engineers.

**Hypothesis 8**

There is a relationship between the number of units the forest engineers had worked and the level of their exposure to bullying.

As stated earlier, studies concerning workplace bullying indicate that the frequency of this phenomenon varies in different sectors and countries. Economic development does not start at the same time in all regions of Turkey, just as is the case with many other countries, and as a result, development focuses on certain centers, thus resulting in different development levels. There are differences between the regions of a country, just as there are socioeconomic and geographic differences between countries [79]. There are 7 geographical regions in Turkey (Mediterranean, Eastern Anatolia, Aegean, Southeastern Anatolia, Central Anatolia, Black Sea, and Marmara), and there are great differences between the index values of the socioeconomic development of the geographical regions. The most developed region of Turkey is the Marmara region, and the least developed region is the Eastern Anatolia Region [80, 81]. Therefore, Hypothesis 9 was made in order to determine the relationship between the levels of exposure to bullying of forest engineers who work at the Regional Directorates of Forestry within these regions and regional differences.

**Hypothesis 9**

There is a relationship between the geographical locations of the Regional Directorates of Forestry and the level of exposure to bullying of their forest engineers.

## 4. Methods

### 4.1. Participants and Procedure

The research was conducted at 27 Regional Directorates of Forestry that are under the aegis of the Ministry of Forestry and Water Affairs. The questionnaire was filled in by 845 forest engineers, 10 of whom were excluded from the research due to insufficient data. Therefore, all the analyses and evaluations in the study were based on 835 questionnaires. The questionnaires were filled in during face-to-face interviews. Of the participants, 81.3%  (*n* = 679) of the participants were male, while 18.7%  (*n* = 156) were female, and 82.9% of all the participants were married; 44.4% were in the 34–44 age group. Of the participants, 16.9% were postgraduates (130 people had a master's degree and 11 people had a doctor's degree), and 31.3% had been working at the Regional Directorates of Forestry for 21 years. Twenty-two percent of the participants were managers. The forest engineers were found to have worked at two different units within the last 10 years, and they had changed their workplace approximately 4 times over the course of their professional lives; two of those occasions involved compulsory redundancies.

### 4.2. Measurements

#### 4.2.1. Bullying at Work

The revised version of the Negative Acts Questionnaire (NAQ-R) [49, 82], which consisted of 22 questions, was used in order to evaluate bullying within the institution considering the characteristics of the research group. Moreover, three additional questions were asked besides those in the NAQ-R in order to determine the frequency of exposure to bullying within the institution and who performs these behaviors. A five-point Likert scale was used to determine the frequency of exposure to both mobbing (never, rarely within the last 6 months, at least once every month, at least once every week, at least once every day) and bullying (every day, every week, every month, rarely, never) within the last 6 months. The Cronbach's *α* value of the NAQ-R was calculated to be 0.92. 

## 5. Results

The main factor groups were formed in order to easily comprehend and interpret the relationship between the questions asked. Therefore, 22 questions were used in the factor analysis. The factor structure of this instrument was analyzed using principal components analysis with varimax rotation. At the end of the analysis, four factors were determined whose eigenvalues were above 1.00 and which explained 54% of the total variance. The factor structure and loadings are given in Table 1. Only the variables whose factor loadings were ≥.40 were included to maximize factor interpretability. 

The factor structures occurring at the end of the analysis were similar to the bullying behavior types determined and examined by Cowie et al. [83]. According to Table 1, questions regarding the individual were examined under factor one, questions regarding the job/duties of the individual were examined under factor two, questions regarding the physical violence or verbal threats were examined under factor three, and questions regarding humiliation were examined under factor four. The Cronbach's *α* values of the factor groups were 0.84, 0.79, 0.68, 0.73, respectively.

### 5.1. Testing the Hypotheses

The *t*-test and ANOVA test were used in order to test the above-mentioned hypotheses. Moreover, crosstabs and frequency tables are also used. The *t*-test results for Hypotheses 1 and 6 are shown in Table 2. 

According to the results of the analysis, a significant relationship was found between gender and humiliation *t*(−2.32) = 0.021, *P* ≤ 0.05. This finding indicated that female forest engineers were more exposed to humiliation compared to male forest engineers. 

ANOVA test was used in order to test Hypotheses 2-3-4-5 and Hypotheses 7-8-9, and the results of the analysis are shown in Table 3. According to the results, a significant relationship was found between age, relevant to person *F*(3,829) = 2.79, *P* ≤ 0.05, and tasks-related behaviors *F*(3,829) = 4.40, *P* ≤ 0.05 in the testing of Hypothesis 2. This finding indicated that people in the 34–44 age group were more exposed to “relevant to person” and “task related” behaviors. 

No significant relationship was found between the marital status and the levels of exposure to bullying of the forest engineers participating in the study, thus resulting in the rejection of Hypothesis 3.

A significant relationship was found between education level and humiliation *F*(2,831) = 4.60, *P* ≤ 0.05 in the testing of Hypothesis 4. This finding indicated that forest engineers with a doctor's degree were more exposed to humiliation compared to those with a bachelor's or a master's degree. That is, exposure to humiliation increases parallel to the education level.

No significant relationship was determined between the duration of professional life and exposure to bullying, thus resulting in the rejection of Hypothesis 5. *t*-test was used to test Hypothesis 6. According to the results, a significant relationship was determined between in-house position *t*(2.70) = 0.007, *P* ≤ 0.05, tasks-related behaviors and *t*(1.95) = 0.05, *P* ≤ 0.05, and humiliation. Managers were less exposed to task-related behaviors and humiliation. A significant relationship was determined between the change of workplace through compulsory redundancy of the forest engineers *F*(2,792) = 9.23, *P* ≤ 0.05, relevant to person behaviors, *F*(2,792) = 10.90, *P* ≤ 0.05, physical violence/verbal threat, and *F*(2,792) = 3.54, *P* ≤ 0.05, humiliation in the testing of Hypothesis 7. That is, exposure to “relevant to person” behaviors and physical violence/verbal threat increases parallel to the number of changes of workplace. A significant relationship was found between the number of units worked and relevant to person behaviors *F*(7,762) = 2.36, *P* ≤ 0.05, in the testing of Hypothesis 8. That is, exposure to relevant to person behaviors increases parallel to the number of units worked.

A significant relationship was found between task-related behaviors, physical violence/verbal threat, humiliation, and geographical location in the testing of Hypothesis 9; however, there was no statistically significant relationship between the above-mentioned factors and relevant to person behaviors. The Eastern Anatolia region was found to be the most exposed to task-related behaviors and physical violence/verbal threat, while the Marmara region was found to be the most exposed to humiliation.

Of the participants, 47.9% stated that they rarely witnessed the exposure of their colleagues to such behaviors. Similarly, 49.2% of the engineers stated that they rarely witnessed other workers being exposed to such behaviors. 

## 6. Discussion

Factor structures occurring at the end of the factor analysis of the NAQ-R questions were similar to the ones in other studies conducted in this field [1, 49]. Factor analysis enabled a more comprehensive examination of the bullying behaviors. Moreover, analyses of variance enabled the examination of the reasons for exposure to such behaviors. 

There are many studies examining the relationship between bullying behaviors and gender. Some of these studies stated that there was no difference between the percentages of exposure to bullying as experienced by men and women [73], while some of the studies stated that the percentages of exposure to bullying as experienced by men and women varied according to gender [84]. However, unlike the findings of Moroschan et al., the present study determined that female forest engineers are more exposed to bullying compared to male ones [85]. In Turkey, the Regional Directorates of Forestry are male-dominated institutions. In America, 36% of women stated that they were victims of bullying in male-dominated institutions, while this rate was only 5% in Norway [31]. In the present study, this rate is 23%. Vartia and Hyyti suggested that women are most exposed to derisive talk and sarcastic behavior by their coworkers and managers [86]. Exposure to bullying increases parallel to the ages of the engineers. Similarly to the study of Rayner and Hoel [87], the present study suggests that young engineers are more exposed to bullying compared to the older ones. 44.5% of the young engineers stated that they felt ignored and neglected. This situation is considered to end when professional experience increases. No significant relationship was found between the marital statuses of the forest engineers and their levels of exposure to bullying. Carnero et al. [23] stated that education level affects bullying behaviors, and Özarallı and Torun [88] suggested that exposure to bullying increases parallel to education levels. 36.6% of the engineers with a Ph.D. degree stated that they were most exposed to the undesired “fun and games” of people they had problems with. This finding may be explained by the fact that the colleagues of the people with higher education levels are envious of them. It is quite common in Turkey that unqualified people are appointed to high-level duties through political maneuvering, and most of the time they experience difficulties in building their authority within the institution. This situation has a negative effect on the relationship between the management and the workers. Such managers are found to be insensitive towards the problems of their personnel and experience difficulties in managing the political desires of the workers within the institution [89]. The finding of the present study which determined a relationship between in-house position and bullying also supports this view. Tehrani [90] suggested that managers (task related: 27.5%; humiliation: 16.7%) were less exposed to bullying compared to the workers (task related: 32.8%; humiliation: 20.6%). In forestry organizations, women are generally not appointed as managers, and thus they are more exposed to bullying compared to their male colleagues. Toksoy et al. [89] suggested that units worked in and changes of workplace occur quite often in the forestry sector in Turkey. Similarly, the present study determined that only 4% of the forest engineers had never changed the unit he/she worked in and only 4.2% had never changed his/her workplace. Moreover, the frequency of exposure to bullying increases parallel to the number of units and workplaces worked. As a result, the engineers feel themselves to be worthless most of the time they work in particular institutions, and this decreases the productivity of those institutions. 

Studies concerning Turkish culture are very important in the analysis of the evaluations regarding the relationship between bullying behaviors and the geographical locations of the Regional Directorates of Forestry. In a study examining the culture level value dimensions of 34 cultures, Turkey ranked (12th), (5th), (16th), and (13th) in regard to conservatism, hierarchy, harmony, and egalitarian commitment, respectively [91]. Similarly, as a result of the GLOBE study evaluating 62 countries, Turkey ranked (10th), (56th), (45th), (12th), (37th), (4th), (42th), and (49th) in regard to power gap, gender egalitarianism, performance orientation, assertiveness, humane orientation, in-group collectivism, societal collectivism, and uncertainty avoidance in terms of intercountry societal culture values, respectively [92]. 

As a result of the analysis of the relationship between bullying behaviors and the geographical locations of the Regional Directorates of Forestry, bullying behaviors were determined to be the most common in the Eastern Anatolia region with 28.7% and to be the least common in the Aegean region with 19.9%. Tasks-related behaviors were found to be the most common in the Eastern Anatolian region with 44.7% and to be the least common in the Central Anatolia region. Of the engineers working in the Eastern Anatolia region, 66.7% stated that they were asked to perform unreasonable or time-limited duties that were impossible to complete. Physical violence/verbal threat behaviors were found to be the most common in the Eastern Anatolia region with 20.2% and to be the least common in the Black Sea region with 12.9%. Of the engineers working in the Eastern Anatolia region, 36.7% stated that they were exposed to unfounded sudden anger or fury at the workplace, and were yelled at for no reason. Humiliation was found to be the most common in the Marmara region, Turkey's most developed region, with 26.2%, and to be the least common in the Black Sea region with 16.2%. Of the engineers working in the Marmara region, 34.4% stated that they were subjected to excessive supervision beyond the normal standards. 

As the above-mentioned studies indicate, there are many issues that need to be developed in Turkey regarding cultural values. The findings of the present study indicate that bullying behaviors in the Regional Directorates of Forestry support this view, and the main reason for the differences in the bullying behaviors that the forest engineers are exposed to is the place of the above-mentioned values in Turkish culture and the regional differences in these values in Turkey. 

## 7. Conclusion

The present study examines the relationship between the demographic characteristics of forest engineers working in branches of the forestry organization in Turkey, the geographical location of the region where they work and their exposure to bullying. For this purpose, hypotheses were made and statistical analyses were conducted. The results of the analyses in the present study support the results of similar studies in the literature. However, the fact that Regional Directorates of Forestry are public institutions, that the victims of bullying hide their exposure to bullying, so as not to gain a “bad” reputation within the institution, and that forest engineering is a profession performed in natural settings, where the engineers spend most of their time in the forests, resulted in lower bullying rates than expected. Bullying rates vary in accordance with the development levels of the regions, which results from the regional cultural differences. All the results obtained through the present study represent the forestry organization in Turkey, and further studies covering various public institutions should be conducted in order to make any generalization concerning such institutions. Moreover, these studies should include a consideration of manager types, the work ethic, and the legal aspects of the issue. 

## Figures and Tables

**Table 1:** Factor analysis for bullying instrument.

	Relevant to person	Task related	Physical violence/verbal threat	Humiliation
(1) Did anyone at your workplace hide information from you which you believe would affect your success?	.517			
(5) Did anyone ever spread an unfounded rumor about you or gossip about you?	.488			
(6) Have you ever felt that you were ignored or excluded or that you were treated as if you were worthless?	.772			
(7) Have you ever been insulted regarding your personality, your attitudes, your private life, or your values?	.488			
(12) Have you ever been ignored, neglected, or mistreated by your coworkers?	.750			
(13) Were your work activities or projects subjected to unfounded criticism?	.555			
(14) Have you ever felt that your ideas and opinions were neglected?	.710			
(3) Have you ever been forced to perform duties beneath your experience, capacity, and education level? Were you ever asked to do such work?		.549		
(4) Have you ever been assigned insignificant, unwanted, and undesirable tasks other than those which are your own responsibility and your task?		.491		
(11) Have you ever been reminded of your previous mistakes in respect of the job?		.589		
(16) Have you ever been asked to perform unreasonable or time-limited tasks that are impossible to complete?		.721		
(19) Have you ever had a heavy workload that you could not manage?		.514		
(21) Have you ever been forced into not claiming your legal rights (annual leave, sick leave, travelling expenses, etc.)?		.719		
(8) Have you ever been exposed to unfounded sudden anger or fury at the workplace? Have you ever been yelled at for no reason?			.455	
(9) Did anyone ever make a threatening gesture towards you? Or have you ever been pushed, physically blocked, or exposed to other such physical behaviors?			.702	
(10) Has it ever been implied that you should quit the job?			.599	
(17) Have you ever experienced serious denunciation, accusations, or incrimination?			.522	
(22) Have you ever experienced ill-treatment or physical or sexual harassment?			.558	
(2) Have you ever been insulted or humiliated regarding your performance?				.453
(15) Have you ever been exposed to undesired “fun and games” by people you have problem with?				.756
(18) Have you ever been subjected to excessive supervision beyond the normal standards?				.690
(20) Have you ever been exposed to derisive conversations, verbal abuse, or sarcasm?				.563

KMO: .940; approx. Chi-square: 6332.073; df: 231; *P* < 0.001; Cronbach alpha: .92.

**Table 2:** *t*-test results for Hypotheses.

Variables	Component											
	Relevant to person			Task related			Physical violence/verbal threat			Humiliation		
	df	*t*	Sig	df	*t*	Sig	df	*t*	Sig	df	*t*	Sig
Gender	833	1.36	0.17	833	0.39	0.69	833	−0.99	0.32	833	−2.32	**0.02**
In-house position	830	0.50	0.61	830	2.70	**0.007**	830	1.33	0.18	830	1.95	**0.05**

**Table 3:** ANOVA results for hypotheses.

Variables	Component															
	Relevant to person				Task related				Physical violence/verbal threat				Humiliation			
	*F*	df1	df2	Sig	*F*	df1	df2	Sig	*F*	df1	df2	Sig	*F*	df1	df2	Sig
Age	2.79	3	829	0.40	4.40	3	829	**0.04**	1.92	3	829	0.12	0.80	3	829	0.49
Marital status	0.48	2	831	0.63	1.55	2	831	0.21	0.48	2	831	0.62	2.15	2	831	0.12
Education level	1.30	2	831	0.27	1.70	2	831	0.18	2.68	2	831	0.07	4.60	2	831	**0.01**
Duration of the professional life	1.35	4	825	0.25	2.32	4	825	0.55	0.62	4	825	0.65	0.56	4	825	0.69
Change of the workplace	9.24	2	792	**0.00**	0.97	2	792	0.38	10.90	2	792	**0.00**	3.54	2	792	**0.03**
The number of units worked	2.36	7	762	**0.02**	0.85	7	762	0.55	1.40	7	762	0.20	2.78	7	762	**0.007**
Geographical location	1.44	6	833	0.19	4.97	6	833	**0.00**	2.66	6	833	**0.01**	3.12	6	833	**0.005**
